# The emergence of non-albicans candidemia and evaluation of HiChrome Candida differential agar and VITEK2 YST® platform for differentiation of *Candida* bloodstream isolates in teaching hospital Kandy, Sri Lanka

**DOI:** 10.1186/s12866-019-1518-3

**Published:** 2019-06-21

**Authors:** Mahen Kothalawala, J. A. A. S. Jayaweera, Sinnapoo Arunan, Anuradha Jayathilake

**Affiliations:** 1 Department of Microbiology, Teaching Hospital Kandy Sri Lanka, Kandy, Sri Lanka; 2 Faculty of Medicine and Allied Sciences, Rajarata University of Sri Lanka, Saliyapura, Sri Lanka

**Keywords:** Candidemia: non-albicans candida sp., Emergence, Risk factors and anti-fungal resistance

## Abstract

**Background:**

Candidemia is an emerging hospital-acquired bloodstream infection (BSI). It is common among severely ill and immunocompromised patients. Even following appropriate therapy in candidemia, recent studies reveal relative high mortality (40%). The global incidence of candidemia shows an incline. In Sri Lanka, *candida* speciation often difficult where basic facilities are less available. We have compared the risk factors, epidemiology, demography, and performance of HiChrome Candida differential agar (HiCA) characteristics with the VITEK2 YST platform for differentiation of *Candida albicans* (CA) and non-albicans *candida* (NAC) from blood culture isolate.

**Methods:**

This is a laboratory-based cross-sectional study. Positive aerobic BACTEC blood cultures having yeast were identified using HiCA and VITEK2® platform. Epidemiology, risk factors, and clinical outcomes were compared between CA and NAC bloodstream isolates.

**Results:**

Out of 120 positive yeast samples, VITEK2® has identified 110 (92%) as *Candida* sp. From that CA*-*34 (31%) and NAC-76 (69%) were isolated. Candidemia following NCA in neonates (*p* = 0.02), infants (*p* = 0.04) and adults (*p* = 0.02) in ICU and immunocompromised patients were significantly higher. Compared to CA, NAC bacteremia period prevalence (0.00041%) and incidence (0.23 per 100,000-person-years) was significantly high (*p* = 0.03). NAC 48 (63%) isolates were resistance to azoles. Exposure to antifungals (odds ratio (OR); p = 0.03), prolonged intensive care stay > 14 days (OR-3.3; *p* = 0.04), having a central venous line for > 8 days (OR-4.3; *p* = 0.03) and on immunosuppressive treatment (OR-2.4; *p* = 0.04) was significantly poses risk for NAC candidemia. Sen day mortality was significant among non-albicans cases (*p* = 0.03) while 30-day mortality was significant among albicans cases (*p* = 0.04). Compared to VITEK2®, the HiCA method was 93% sensitive and 93% specific in detecting CA.

**Conclusion:**

Compared to CA, candidemia following NAC was high. NAC isolates were having a high percentage of fluconazole and voriconazole resistance. VITEK2 YST® platform provides antifungal susceptibility with minimal inhibitory concentration (MIC). Impact, this would highlight the use of rapid *candida* identification flat form with MIC to direct appropriate antifungals for candidemia. For that implementation of novel diagnostic facilities like the VITEK2 YST platform at a tertiary care facility is demanding.

## Background

Candidemia is an emerging nosocomial bloodstream infection (BSI) and is commonly detected in debilitated and immunocompromised patients [1]. Unless persistence of candidemia without appropriate antifungal therapy is invariable ends up in clinical failure and death. Even candidemia is following appropriate therapy, in recent studies reveal it has relatively high mortality (40%) [2].

The incidence of candidemia has incline globally. Similarly, anecdotal reports on candidemia in Sri Lanka show an upward trend. In United State of America (USA) a rate of 72.8 cases/ million candidemia cases were recorded in the year 2015, while in United Kingdom (UK) rate remains around 15.2 cases/million population [3]. At present in the globe, *Candida* spp. other than *C. albicans* (*C. parapsilosis*, *C. glabrata*, *C. krusei,* and *C. tropicalis*) are emerging as opportunistic pathogens. Perhaps, *C. parapsilosis* is the second most frequently isolated *Candida* spp. from blood cultures in Europe, Latin America and Canada [4, 5].

The increased incidence of candidemia has created many health issues including the need for close monitoring with prolonged use of costly and toxic antifungals. The cost for managing a single episode of the candidemia is relatively higher than an episode following bacteremia. Apart from high cost, many other aspects of care need to be addressed as candidemia could be complicated with endophthalmitis and infective endocarditis [6]. As early as a possible patient with candidemia such complications need to exclude. Another hand treatment of candidemia is complicated. Antifungal therapy requires property and performance of antifungal susceptibility testing (AFS) which is time consuming, laborious and can perform only in reference laboratories. Also, compared to antibiotics availability of antifungals in underdeveloped countries is low [7, 8].

Sri Lanka, in most clinical microbiology laboratories fungal speciation often difficult where basic facilities are often less available. *C. albicans* can be detected using germ tube test but differentiation of non- albicans species is difficult and clinician tend to use more toxic drugs (amphotericin B) instead of fluconazole [9, 10]. Perhaps, this practice would lead to the development of resistance among amphotericin B sensitive isolates. A rapid and simple test that can be performed at clinical settings is highly recommended. Using growth characteristics of different candida species on common differential Candida media (chrome agar) is available in few laboratories. In this study, we have compared the risk factors, incidence, age and attended unit prevalence of *Candida albicans* and non-albicans *candida* spp. and performance of HiCrome Candida differential agar characteristics with VITEK2 YST platform for differentiation of *Candida albicans* and non-albicans *candida* spp. from blood culture isolates.

## Results

### Demography and clinical characteristics of patients with candidemia

Over the study period, 12,000 blood cultures were received to the department laboratory. From that 1200 (10%) became positive. Based on gram staining, 120 blood culture samples suspecting to have yeast were included in the study. From that VITEK2 YST® identified 110 (91.6%) samples as candidemia. Out of 110 candidemia cases, 34 (31%) *C. albicans* and 76 (69%) of non-albicans were detected. Candidemia was detected in 9.1% (110 out of 1200) overall bloodstream infections. In both albicans and non-albicans, associated candidemia was having a bimodal age distribution. Among albicans associated candidemia it was 0.9 ± 0.3 years and 68 ± 4.7 years while among non-albicans candidemia was 1.1 ± 0.4 years and 63 ± 6.7 years. In both candidemia cases, male predominance was observed (*p* = 0.04).

### Distribution of albicans and non-albicans candidemia cases

**Fig. 1:**
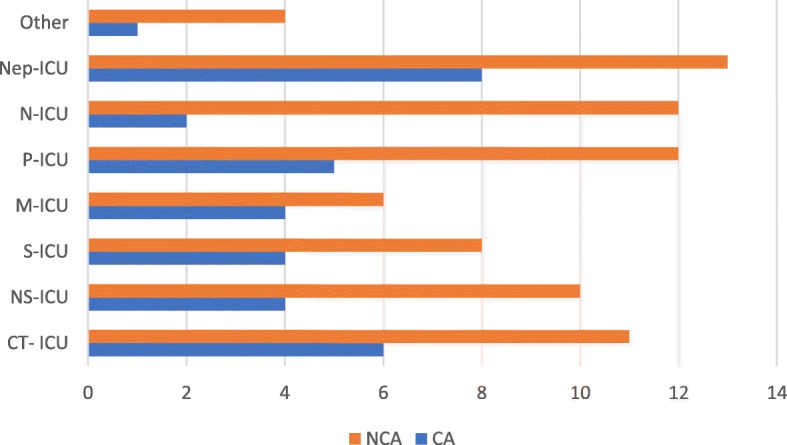
Unit wise distribution of albicans and non- albicans candidemia cases in teaching hospital Kandy ICU- intensive care unit; Nep- nephrology; N-neonatal; P- pediatric; M- medical; S- surgical; NS- neurosurgical; CT- cardiothoracic; NCA- non albicans candidemia and CA- *Candida albicans* candidemia

Candidemia following non-albicans species was significantly higher in intensive care units (ICU) (*p* = 0.03) Also, in plastic surgery and medical wards non-albicans candidemia was high (*p* = 0.04) (Fig. 1).

### Speciation and antifungal susceptibility of candida isolates

**Fig. 2:**
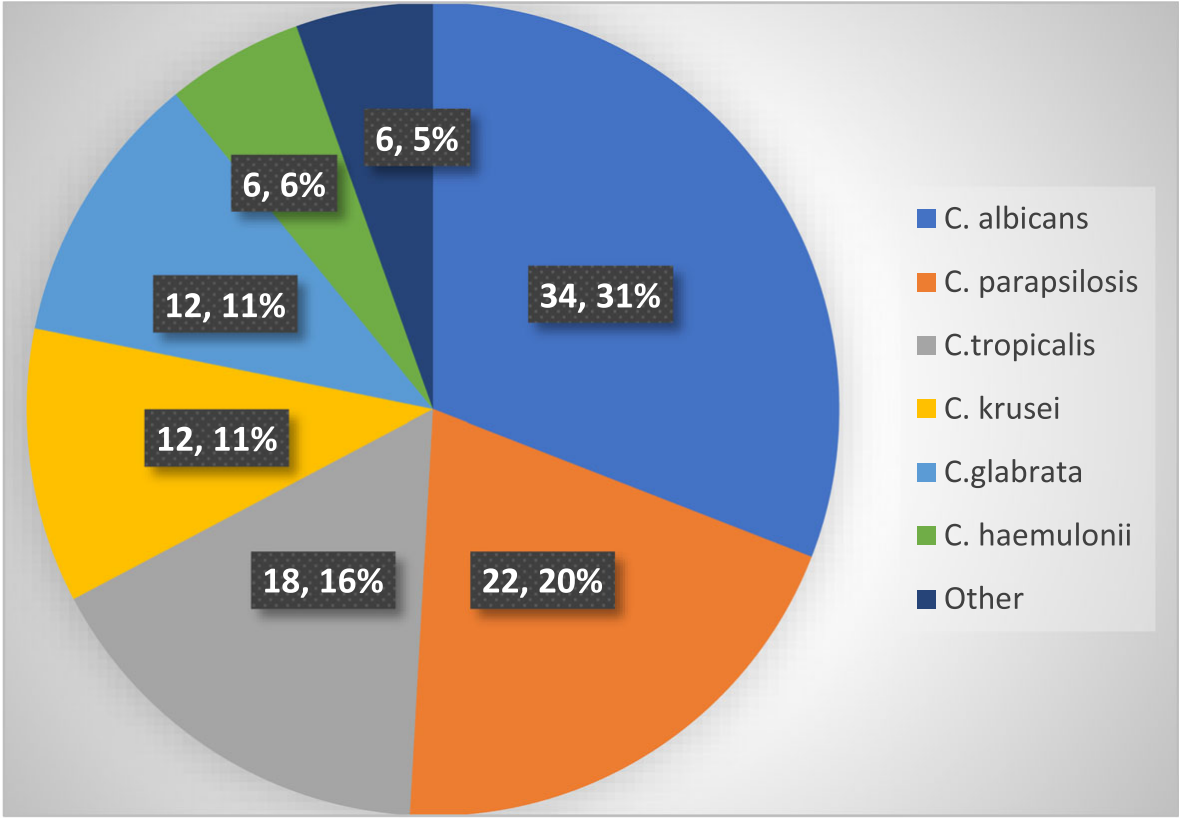
Distribution of Candida species among blood culture isolates (n, %)

When considering a single species *C. albicans* was the most common etiological agent responsible for candidemia and it was isolated in 34 (31%) cases. Overall non- albicans spp. responsible for causing 76 (69%) cases of candidemia. *C. parapsilosis* 22 (20%), *C. tropicalis* 18 (16.4%), *C. glabrata* 12 (11%), *C. krusei* 12 (11%), *C. haemulonii* 6 (5.5%) and other (2- *C. intermedia*, 2- *C. lusitaniae* and 2- *C. rugosa*) non-albicans was isolated in 6 (5.5%) cases following candidemia (Fig. 2).

Based on VITEK2 YST® 6 out 34 *C. albicans* isolates were resistant to fluconazole and minimal inhibitory concentration (MIC) ranged from 2 to 16 μg/ml (< 8 μg/ml is taken as susceptible). Further, 4 out of 6 above isolates were resistant to voriconazole as well. Three out of 6 germ tube negative *C. albicans* (50%) were having fluconazole and voriconazole resistance. All together non-albicans poses 63% (48/76) resistance to azoles. Compared to CA azole resistance was significantly higher among NAC (*p* = 0.02). Twelve out of 22 *C. parapsilosis* isolates and 10 out of 18 *C. tropicalis* isolates were resistant to fluconazole and MIC ranged from 8 to 64 μg/ml (< 8 μg/ml is taken as susceptible). Further, 2 out of 4 above isolates were resistant to voriconazole as well. All *C. glabrata, C. lusitaniae, C. haemulonii*, and *C. krusei* were resistant to both fluconazole and voriconazole while *C. intermedia* and *C. rugosa* were susceptible to fluconazole and voriconazole. All *Candida* spp. except *C. lusitaniae* was sensitive to amphotericin B.

### *Candida* spp. period prevalence and incidence among different groups of patients

The period prevalence and incidence of different *Candida* spp. (including albicans and non-albicans) overall and different patient categories were displayed in Table 1. Overall, *C. albicans* fungemia having the highest period prevalence and incidence and it was 0.00017% and 0.113 per 100,000-person-years respectively. Further, period prevalence and incidence were highest among neonates, infants, adults in ICU and immunocompromised patients from oncology, nephrology and in ICU in THK. Also, *C.* parapsilosis*, C. tropicalis, C. krusei,* and *C. glabrata* w*ere* detected in all patient categories while *C. haemulonii* was not detected in neonates in ICUs. When compared to *C. albicans* the incidence and period prevalence of non-albicans *Candida* spp. among neonates (*p* = 0.02), infants (*p* = 0.04) and adults (*p* = 0.02) in ICU and immunocompromised patients from oncology, nephrology and in ICU (*p* = 0.03) were significantly high.

**Table 1:** Incidence and prevalence of isolated *candida* species among different patient categories using VITEK2 YST® platform

Category of patients	Candida isolates in blood culture from VITEK2 YST® platform	Number of cases	Period prevalence (%)	Incidence/100,000-person years	Comments and *p* value
Overall	*C. albicans*	34	1.7 X 10 ^−4^%	0.113	0.03 non- albicans period prevalence and incidence was significantly higher than candidemia following albicans
	*Non- albicans*	76	4.1 X 10 ^−4^%	0.23	
	*C. parapsilosis*	22	1.1 X 10 ^−4^%	0.07	
	*C. tropicalis*	18	1.0 X 10 ^−4^%	0.06	
	*C. krusei*	12	8 X 10 ^−5^%	0.04	
	*C. glabrata*	12	8 X 10 ^− 5^%	0.04	
	*C. haemulonii*	6	4 X 10 ^−5^%	0.02	
	Other	6	4 X 10 ^−5^%	0.02	
Neonates in intensive care units	*C. albicans*	2	2 X 10 ^−3^%	0.133	0.02 non- albicans period prevalence and incidence was significantly higher than candidemia following albicans
	*Non- albicans*	8	9 X 10^−3^%	0.6	
	*C. parapsilosis*	1	2 X 10 ^−3^%	0.133	
	*C. tropicalis*	3	3 X 10 ^−3^%	0.2	
	*C. krusei*	1	2 X 10 ^−3^%	0.133	
	*C. glabrata*	2	2 X 10 ^−3^%	0.133-	
	*C. haemulonii*	0			
Infants in intensive care units	*C. albicans*	6	3 X 10–3%	0.2	0.04 non- albicans period prevalence and incidence was significantly higher than candidemia following albicans
	*Non- albicans*	7	6 X 10–3%	0.4	
	*C. parapsilosis*	1	2 X 10–3%	0.133	
	*C. tropicalis*	3	1.5 X 10–3%	0.1	
	*C. glabrata*	2	1.5 X 10–3%	0.066	
	*C. krusei*	1	1 X 10–3%	0.066	
	*C. haemulonii*	0			
Adults in intensive care units	*C. albicans*	22	1.8 X 10–4%	0.12	0.02 non- albicans period prevalence and incidence was significantly higher than candidemia following albicans
	*Non- albicans*	56	3.8 X 10–4%	0.25	
	*C. parapsilosis*	20	1.4 X 10–4%	0.09	
	*C. tropicalis*	12	9 X 10–5%	0.06	
	*C. glabrata*	9	6 X 10–5%	0.04	
	*C. krusei*	9	6 X 10–5%	0.04	
	*C. haemulonii*	6	3 X 10–5%	0.02	
Immunocompromised patients (having neutropenia^a^)	*C. albicans*	22	1.1 X 10–3%	1.13	0.03 non- albicans period prevalence and incidence was significantly higher than candidemia following albicans
	*Non- albicans*	51	3.4 X 10–3%	1.68	
	*C. tropicalis*	16	8 X 10–4%	0.53	
	*C. parapsilosis*	10	5 X 10–4%	0.33	
	*C. glabrata*	10	5 X 10–4%	0.33	
	*C. krusei*	10	5 X 10–4%	0.33	
	*C. haemulonii*	5	1.1 X 10–5%	0.16	

^a^Neutropenia – neutrophil count < 500 / mm^3^; *p* < 0.05 taken as significant

### Risk factors and clinical outcome of albicans and non-albicans candidemia cases

Based on VITEK2 YST® identification platform comparison of risk factors and treatment outcome of patients with *C. albicans* vs. non-albicans candidemia was displayed in Table 2.

**Table 2:** Risk factors and treatment outcome of patients with candidemia in Teaching Hospital Kandy

	Candidemia (*n* = 110)		
Parameters	*C. albicans* (*n* = 34)	Non albicans (*n* = 76)	Odds ratio confident interval (95%) and *P* value
Age	–	–	> 0.05
Gender male: female	1.2:0.8	1.3:0.7	> 0.05
Clinical risk factors			
Diabetes	26 (23.6%)	19 (25%)	> 0.05
Liver failure	12 (11%)	19 (25%)	> 0.05
Chronic lung diseases	15 (13.6%)	16 (21%)	> 0.05
Renal failure	12 (35.2%)	18 (24.3%)	> 0.05
Solid organ malignancy	7 (20.6%)	12 (16.2%)	> 0.05
Hematological malignancies	6 (17.6%)	16 (21%)	> 0.05
Exposure of broad spectrum antibiotics	28 (82.3%)	56 (73.7%)	> 0.05
Exposure of anti-fungal	3 (8.8%)	38 (50%)	2.2 (1.9–3.2); 0.03
Under gone abdominal surgeries	7 (20.6%)	19 (25%)	> 0.05
On immune suppressive medications	6 (17.6%)	38 (50%)	2.4 (2.1–2.9); 0.04
On hemodialysis	12 (35.2%)	22 (29.7%)	> 0.05
Prolonged intensive care stay ≥14 days	8 (23.5%)	56 (73.7%)	3.3 (2.2–4.5); 0.03
On central venous line > 8 days	16 (47.0%)	64 (84.2%)	4.3 (3.4–5.4); 0.03
On urinary catheters > 10 days	18 (52.9%)	38 (50%)	> 0.05
Mechanical ventilation			
Presence of prosthesis or implant	12 (35.5%)	26 (35.5%0	> 0.05
Total parenteral nutrition	6 (17.6%)	16 (21%)	> 0.05
Candiduria	6 (17.6%)	16 (21%)	> 0.05
	6 (17.6%)	12 (15.7%)	> 0.05
Duration of treatment	16.8 ± 2.2 days	26.8 ± 2.2 days	0.03
Out come			
Complete recovery	28 (82.3%)	48 (63.1%)	0.04
Death	6 (17.7%)	28 (36.9%)	0.04
Day 7	2 (33.3%)	18 (64.2%)	0.03
Day 30	4 (66.6%)	10 (35.8%)	0.04

*P* < 0.05 taken as significant

After univariate analysis (factors having *p*-value < 0.1 was taken as significant), the following 19 variables were considered to be candidates for the multivariate model: age, gender, preexisting lung disease, liver failure, renal failure, solid organ malignancy, hematological malignancy, undergone renal transplantation, gastrointestinal procedures, use of broad-spectrum antibiotics, use of fluconazole therapy, duration of central venous catheter use (taken when it was > 5 days), duration of urinary catheter use (taken when it was > 5 days), prolonged intensive care unit stay (taken when it was > 14 days), total parenteral nutrition, Candiduria and immunosuppressive therapy.

In the final multivariate model in multivariate logistic regression analysis, exposure to antifungals (odds ratio (OR)-2.2; 95% confidence interval (CI)- 1.9–3.2; *p* = 0.03), prolonged intensive care stay > 14 days (OR-3.3; 95% CI- 2.2-4.5; *p* = 0.04), having a central venous line for > 8 days (OR-4.3; CI- 3.4-5.4; p = 0.03) and immunosuppressive treatment (OR-2.4; CI- 2.1- 2.9; *p* = 0.04) was significantly associated with non-albicans associated candidemia. Among patients who received antifungal therapy before developing candidemia, the majority received fluconazole and a few patients received amphotericin (of any formulation), or voriconazole. No independent associations were found with age, gastrointestinal procedures, having comorbidities including liver failure, renal failure, solid malignancy, hematological malignancy, undergone renal transplantation, Presence of prosthesis or implant and having total parenteral nutrition was not significantly poses risk for development of albicans vs. non-albicans candidemia cases. Similarly, male gender was not significantly posing risk for the development of albicans vs. non-albicans candidemia but overall male gender poses a significant risk for the development of candidemia.

When considered the outcome complete recovery in albicans and non-albicans cases were 28 (82.3%) and 46 (63.1%) respectively. The recovery rate was significantly high among albicans associated candidemia cases (*p* = 0.04). Day-7 mortality was significant among non-albicans cases (*p* = 0.03) while day-30 mortality was significant among albicans cases (*p* = 0.04). The day-7 and day-30 mortality were not significantly associated with the age and gender of patients. Patients with non-*albicans* associated candidemia were having a significantly longer duration of hospitalization (*P* = 0.03).

### Comparison between the VITEK2 YST platform and HiChrome Candida differential agar for Candida identification and speciation

HiChrome Candida differential agar is only capable to identify *C. albicans, C. tropicalis, C. glabrata* and *C. krusei*. HiChrome Candida differential agar sensitivity and specificity for *C. albicans* were 93 and 93% respectively. Further for *C. tropicalis* sensitivity was 96% while specificity was 95%. *C. glabrata* and *C. krusei* sensitivity was 86% & 82% while specificity was 90% & 90% respectively. Also, VITEK2 YST flat form is capable of identifying other non-albicans species including *C. parapsilosis*, *C.* lusitaniae*, C. intermedia, C. haemulonii*, *and C. rugosa.*

### HiChrome Candida differential agar and VITEK2 YST® platform

Also, the VITEK2 YST® platform is capable of identifying *Cryptococcus neoformans* (4 isolates). Also, rare but medically important fungi like *Trichosporon asahii* (4 isolates) and *stephanoascus ciferrii* (2 isolates). Based on HiChrome Candida differential agar these 10 isolates were misidentified as *Candida* spp.

## Discussion

In our study, candidemia was detected in 91.6% following fungal and in 9.1% (110 out of 1200) overall bloodstream infections. In the USA, it is among the top five pathogens causing nosocomial bloodstream infections (BSIs) and *Candida* species cause 8 to 10% of nosocomial BSIs [11, 12]. Among candidemia patients, male predominance and bimodal age distribution were observed where it was common in patients < 1 year and > 60 years of age. This is compatible with a study done in Iceland on 2012 as age-specific incidence rates were highest among patients at the extremes of age, 20.7/100,000 for < 1 year of age and 18.1/100,000 for > 60 years [13].

Although *C. albicans* remains the predominant etiology of fungal BSIs, recent epidemiologic studies of candidemia have demonstrated an increased incidence of infections due to non-*albicans Candida* species in the United States and Europe [14, 15]. In our study, it was 69% (0.23/ 100,000-person-years) while a recent study in India reveals isolation of non-Candida species increased from 0.633 in 1999 to 9.38 episodes/ 10,000 patient days in 2008 with the rate of change over time is 28.9%, which was significant [16]. In Japan showed more frequent isolation of *Candida* spp. following peripheral line associated candidemia (8.1 to 14.7%) [17].

In this study candidemia, the overall prevalence was 0.0004% and incidence was 0.35 per 100,000-person-years. On 2008–2013 multicentered study in the USA showed candidemia incidence ranged from 9.5–30.9 per 100,000-person-years [18]. Two multicenter epidemiological studies over 2008–2009 and 2009–2010 in tertiary care hospitals in Spain candidemia incidence in 2008–2009 and 2009–2010 was 109 cases/100,000 admissions and 92 cases/100,000 admissions, respectively [19]. Following multicenter studies in Italy rise of candidemia, the rate was reported from 1999 to 2009. The candidemia rate was found as 119 cases/100,000 admissions in 2009, while this rate was 38/100,000 admissions in the 1997–1999 period [20]. Although, a smaller number of studies on candidemia have been conducted in Latin America. A study conducted between 2008 and 2010 reported a candidemia incidence of 118/100,000 admissions [21]. Here, it was having a relatively low incidence of candidemia. This could be due to having a low number of HIV/AIDS patients and patients with malignancies [22].

The candidemia incidence in ICUs was 1.5 cases per 1000 -person-years. In a 20-year survey from Toronto, Canada in 2004 have shown that the rate of candidemia in the ICU was 0.5 cases per 1000 -person-years. In France, on 2005–2006 and 16 cases per 1000 -person-years and in China it was 32 cases per 1000 -person-years [23]. In India on 2012 candidemia incidence all across ICUs was 65 cases per 1000 -person-years [24]. Our study had a relatively low incidence in ICU and is closer to Canada. Further, 94.5% of candidemia were acquired from ICU whiles only 5.5% acquired from the plastic surgery unit. Data from the SENTRY Antimicrobial Surveillance Program from Europe, North America, and Latin America, showed that 44.5% of candidemia were ICU-acquired over the 2008–2009 period [25]. A relatively high percentage of candidemia prevalence among ICU set up would reflect the risk associated with an ICU stay. In ICUs, depending on a number of days, to reduce the acquisition of *candida* infections clinicians need to revise central lines and indwelling catheters. To further confirm the above results a long term and large sample study is required.

When considered non-albicans, *C. parapsilosis* causes 30% of the candidemia cases among newborns whereas the rate is 10–15% among adults [12]. In our study, it was common among neonates where incidence was similar to *C. albicans* incidence. Also, *C. parapsilosis* was common among infants and adults in ICU. *C. glabrata* is common among adults and also patients with neoplasia [21]. Similarly, in our study, it was isolated from patients with malignancies. *C. tropicalis*, on the other hand, is more commonly seen among leukemia patients and neutropenic patients [12]. In our study, after *C. albicans, C. tropicalis* was common in patients with neutropenia. Since *C. parapsilosis* colonizes the skin, it is a common pathogen in catheter-related infections and may cause outbreaks. Further, *C. krusei* is more common among hematopoietic stem cell recipients or neutropenic leukemia patients receiving fluconazole prophylaxis [4, 11, 21]. In our study, *C. krusei* was isolated from neutropenic patients and was relatively less compared to *C. albicans* and *C. tropicalis.*

In our study, 17.6% of *C. albicans* species were germ tube negative. Studies reveals that > 5% isolates are germ tube negative [26, 27]. Importantly, 50% of germ tube negative *C. albicans* were both fluconazole and voriconazole resistant. Our study, *C. albicans*, 17.6% was fluconazole resistant while 11.1% was voriconazole resistant. Since AFST results are unavailable, depending on germ tube test negativity thus assuming the non-albicans isolate is resistant to fluconazole, clinicians tend to put amphotericin B as first-line treatment. It could lead to a cure but simultaneously this practice would create anti-fungal resistance. Also, cost, as well as associated adverse effects (mainly nephrotoxicity), are higher following amphotericin B. Therefore, following speciation determination of antifungal MIC is crucial.

In our study, exposure to antifungals (OR-2.2; CI- 1.9-3.2), prolonged intensive care stay > 14 days (OR-3.3; CI- 2.2-4.5), having a central venous line for > 8 days (OR-4.3; CI- 3.4-5.4) and immunosuppressive treatment (OR-2.4; CI- 2.1- 2.9) poses higher risk towards development of non-albicans candidemia. The rest of the risk factors being insignificant and it implies that those known risk factors are similar for both albicans and non-albicans associated bacteremia. A Boston study reveals that fluconazole exposure to be a risk factor for the development of candidemia due to non-*albicans Candida* species (OR, 11.6) [28]. Further, A study conducted between 2001 and 2005 in Greece identified steroid use, having a central venous catheter, and Candiduria is independent risk factors for infections due to non-*albicans* species [29]. A similar study conducted in the USA between 1995 and 2005 reported that the duration of fluconazole treatment and central venous catheterization are significant risk factors for the development of candidemia [30]. A study from Australia, which was conducted during 2001–2005 and included 50 ICUs, identified the following as significant risk factors for non-*albicans Candida* infections: previous systematic antifungal therapy, gastrointestinal surgery, old age, and intravenous drug use [31].

When we considered the mortality at day-7 it was high following non-albicans candidemia while day-30 it was high following *C. albicans* associated candidemia. A study in Taiwan reveals that in patients with candidemia, all-cause day 7 mortality rate was 38.0% (41 of 108). Day 7 mortality rates of *C.albicans* and non-albicans were demonstrated as 44.3% (27 of 61) and 29.8% (14 of 47). When we considered the virulence hemolysin and biofilm production was similar while only phospholipase production was better in *C. albicans* in comparison to non-albicans sp. [32]. But having higher mortality could be the following emergence of high virulence among non-albicans candida sp. Further, failure of host-defense mechanisms and to complications associated with the patients underlying disease especially more severely ill patients are at a higher risk of *Candida* infection and have a worse prognosis [33]. This is particularly evident in ICU patients who require indwelling central venous catheter and prolonged ICU stay.

Identification of *candida* species from HiChrome Candida differential agar is based on the formation of various colored colonies which result from the use of chromogenic substrates by species-specific enzymes. These enzymes allow organisms to be identified at the species level by their color and colony characteristics [34, 35]. HiChrome Candida differential agar is only capable in the identification of *C. albicans*, *C. glabrata*, *C. tropicalis*, and *C. krusei.*

VITEK2 YST® platform is capable of detecting most of common as well as uncommon *candida* species. Following isolation, within 4 h, in addition to speciation, it gives MIC for fluconazole and voriconazole [36, 37]. When considered the capital cost, it is expensive. But overall with the continuous workflow with less use of consumables and manpower, it would rather cheap. In contrast, the HiChrome Candida differential agar method, it takes 18 h while the need to manually interpret the outcome through visual comparison between cultures with the provided color code. This could lead to erroneous results and it provides only the identification of 4 common species only [38, 39]. Another hand, with the emergence of non-albicans candida species the use of HiChrome Candida differential agar would be limited. The sensitivity of HiChrome Candida differential agar was ranged from 82 to 96% while specificity ranged from 90 to 95%. Further,

### Limitations

Over the 2 years, we got only a limited number of sample positivity. The study was conducted in a single center. Therefore, having a low number of samples would have reduced additional data analysis.

## Conclusion

Compared to *C. albicans,* the incidence and prevalence of non-albicans candidemia are high and emerging. Non-albicans isolates pose a high level of fluconazole and voriconazole resistance. Further, compared to the chrome agar, the VITEK2 YST® platform provides antifungal susceptibility with MIC. This would guide clinicians to prescribe targeted antifungals. In the future, this would lead to proper patient care with the rational use of anti-fungal with minimal emergence of antifungal resistance.

## Methods

This is a laboratory-based cross-sectional study from May 2015–May 2017 conducted in clinical Microbiology Laboratory, Department of Microbiology, Teaching Hospital. Kandy (THK), Sri Lanka.

### Identification of *candida* species

Aerobic automated BACTEC blood cultures having positive *Candida* isolates were allowed to grow on HiCrome Candida differential agar and identified using reference color cards provided by the manufacturer. HiCrome Candida Differential Agar is a selective and differential medium, which facilitates rapid isolation of yeasts from mixed cultures and allows differentiation of *Candida* species namely *C. albicans, C. krusei, C. tropicalis,* and *C. glabrata* based on coloration and colony morphology [35]. Using this medium result can be obtained within 48 h. The same blood culture isolates were inoculated in blood agar and sabouraud dextrose agar and identified using automated VITEK2 YST platform. VITEK 2 is an automated microbiology system utilizing growth-based technology. A transmittance optical system allows interpretation of test reactions using different wavelengths in the visible spectrum. During incubation, each test reaction is read every 15 min to measure either turbidity or colored products of substrate metabolism. Based on biochemical tests it gives the best match of the isolate from its database. Further, the antifungal susceptibility of each isolate for commonly used antifungals was obtained using the VITEK2 YST automated platform. YST contains 2-fold serial dilutions of amphotericin B (range, 0.03 to 16 μg/ml), fluconazole (range, 1 to 128 μg/ml), and voriconazole (range, 0.125 to 16 μg/ml) [38]. AFS breakpoint was taken from CLSI M27-S4 [40]. For fluconazole MIC ≥8 μg/dl (*C. albicans*, *C. tropicalis* and *C. parapsilosis)*; MIC ≥64 μg/dl (*C. glabrata)* and for voriconazole ≥1 μg/dl (*C. albicans*, *C. tropicalis*, and *C. parapsilosis)*; MIC ≥2 μg/dl (*C. krusei*) was taken as clinical breakpoints. When we use pure culture isolates, the VITEK2 platform gives the identity and the AFS within 4 h. Germ tube was performed to assess the laboratory-based identification of *C. albicans* vs. non-albicans spp.

### Clinical and demography data collection

Clinical data, risk factors, and outcomes were collected by a trained medical officer at patient visits by using a self-administrative questionnaire. All patients on radiotherapy, chemotherapy, solid and hematological malignancies, including patients having absolute neutropenia (< 500/ ml) and end-stage renal failure patients were taken as immunocompromised.

### Study variables

Diseases, including, liver failure, renal failure, solid organ malignancy, hematologic malignancy, undergone renal transplantation, diabetes, and neutropenia, were recorded. Predisposing factors that occurred within 30 days before the onset of candidemia were also collected. These included central venous catheter usage; presence of a prosthesis, implant, or indwelling of a urinary catheter; receipt of mechanical ventilation; receipt of corticosteroids; hemodialysis; gastrointestinal procedures and operations; use of broad-spectrum antibiotics; candiduria; exposure to antifungal agents; and intensive care unit (ICU) stay.

### Statistical analysis

The data were double checked and transported to SAS 9.1 (2005 New Jersey) for statistical analysis [41]. The incidence and prevalence of *Candida* spp. were calculated using standard formulas. Further, sensitivity and specificity were calculated using standard formulas [11]. Continuous data were expressed in measures of central tendency. The chi-squired test/ Fisher’s exact test was performed to compare the chrome agar method and the VITEK2 YST®. Factors associated with candidemia due to non-*albicans Candida* spp., compared with *C. albicans* candidemia, were examined using χ^2^ tests or univariate logistic regression. Variables that had statistical significance at *P* < 0.10 in the univariate analysis were considered to be candidates for the developing multivariable models.

Stepwise and best subsets approaches were used to build multivariate logistic regression models to determine which variables were most strongly associated with BSIs due to non-*albicans Candida* spp. A *P* value of < 0.05 was considered to be statistically significant in multivariable modeling.

## Data Availability

The datasets used and analyzed in the current study are available from the corresponding author on reasonable request.

## Abbreviations

AFS: Antifungal susceptibility testing
BSI: bloodstream infection
CA: *Candida albicans*
CI: Confidence interval
CLSI: Clinical laboratory standard institute
ICU: Intensive care unit
NAC: Non-albicans *candida*
OR: Odds ratio
SAS: Statistic analytical software
THK: Teaching Hospital. Kandy
UK: United Kingdom
USA: United State of America
